# Minimally invasive treatment for anterior pelvic ring injuries with modified pedicle screw-rod fixation: a retrospective study

**DOI:** 10.1186/s13018-018-0945-4

**Published:** 2018-09-17

**Authors:** Chun-Chi Hung, Jia-Lin Wu, Yuan-Ta Li, Yung-Wen Cheng, Chia-Chun Wu, Hsain-Chung Shen, Tsu-Te Yeh

**Affiliations:** 1 Department of Orthopedic Surgery, Tri-Service General Hospital and National Defense Medical Center, 325 Cheng-Kung Road, Section 2, Taipei, 114 Taiwan; 2 Department of Orthopedics, School of Medicine, College of Medicine, Taipei Medical University, Taipei, Taiwan; 3 Department of Orthopedics, Taipei Medical University Hospital, Taipei, Taiwan

**Keywords:** Pelvic ring, Minimally invasive treatment, Modified pedicle screw-rod fixation (MPSRF)

## Abstract

**Background:**

Pelvic ring injuries constitute only 2 to 8% of all fractures; however, they occur in 20% of polytrauma patients. High-energy pelvic fractures often result in mechanical instability of the pelvic ring. Successful treatment of unstable pelvic ring fractures remains a challenge for orthopedic surgeons. This study presents a novel internal fixation method for stabilizing unstable anterior pelvic ring fractures using a minimally invasive modified pedicle screw-rod fixation (MPSRF) technique.

**Methods:**

This retrospective study included six patients with unstable pelvic ring injuries who underwent MPSRF, with or without posterior fixation. Intraoperative parameters such as blood loss, operative time, complications, and quality of reduction (Matta criteria) were recorded and evaluated by a blinded reviewer.

**Results:**

In the present clinical series, the mean operative times and mean blood loss for unilateral versus bilateral anterior ring fixations were 176.0 min versus 295.6 min, and 153.3 mL versus 550.0 mL, respectively. No iatrogenic neuropraxia of the lateral femoral cutaneous nerve or femoral nerve palsy occurred. The reduction quality, graded by the Matta criteria, was excellent in five patients and good in one patient.

**Conclusions:**

There were no infections, delayed unions, nonunions, or loss of reductions during the follow-up period. Only one patient suffered from a broken rod at 4 months postoperatively. The modified technique represents a novel, minimally invasive procedure for the treatment of anterior pelvic ring fractures and offers a reliable and effective alternative to currently available surgical techniques.

## Background

Pelvic ring injuries constitute only 2 to 8% of all fractures but occur in 20% of polytrauma patients [1]. High-energy pelvic fractures often result in mechanical instability of the pelvic ring. Pelvic fixation has traditionally been divided into posterior and anterior fixation, and although pelvic stability is mainly sustained by the posterior ring, the anterior ring provides 30% of pelvic stability [2]. Thus, to acquire better reduction of unstable pelvic fractures, a combination of anterior and posterior fixation is needed.

Stabilization of anterior pelvic ring fractures can be achieved via multiple techniques, including external fixation [3], open reduction and internal fixation with plating, or percutaneous trans ramus screw fixation [4]. External fixation is helpful for initial hemodynamic stabilization, and it involves lower operating time and blood loss than does open surgery. However, there are limitations to this treatment, including pin tract infections, aseptic loosening, hindrance to surgical abdominal access, and difficulties in nursing care [5, 6]. Open reduction has the potential disadvantage of an extensive exposure, which includes muscle stripping, and the risk of damage to neurovascular structures [7].

With the aim of improving patient comfort and minimizing the complications associated with traditional treatment techniques, minimally invasive techniques have been widely used for anterior pelvic ring fixation. The potential benefits include minimal soft tissue dissection, diminished surgical site infections, and faster patient rehabilitation with better pain control. These procedures comprise subcutaneous implants fixed into the ilium with or without fixation into the parasymphyseal region (reported as the pelvic bridge) [8], the occipitocervical spinal plate-rod technique [9], and an anterior subcutaneous pedicle screw-rod internal fixator (INFIX) [10–12]. However, iatrogenic lateral femoral cutaneous nerve (LFCN) palsy is a common complication of these procedures and is reported in 30 to 48.3% of patients [13, 14]. The placement of pedicle screws in the supra-acetabular region, as in external fixation, and of INFIX, requires incisions directly over the anterior inferior iliac spine (AIIS) with pedicle screws placed in a high-risk zone for LFCN [15].

Inspired by the pelvic bridge with the plate-rod fixator and INFIX techniques, we designed a new method in which a submuscular pedicle screw-rod device was placed through small incisions over the iliac wing and the pubic region. We modified the pedicle screw position from the AIIS to being over the inner table of the iliac bone and the ipsilateral or contralateral superior pubic ramus, which was dependent on the fracture pattern, fixed with connecting rods. We aimed to evaluate the clinical application of this minimally invasive modified pedicle screw-rod fixation (MPSRF) technique for the treatment of anterior pelvic ring fractures.

## Methods

This retrospective clinical series included patients who presented to the Tri-Service General Hospital, a level 1 trauma center in Taiwan, between October 2014 and October 2016. Six patients with unstable pelvic ring injuries underwent anterior fixation using the MPSRF technique, with or without posterior fixation. If posterior ring instability was present, it was first operated on using standard techniques of reduction and fixation methods, such as percutaneous iliosacral screws, percutaneous transiliac plates, or spinal pelvic fixations. The exclusion criteria were (1) hemodynamically unstable patients, (2) infections or soft tissue defects, (3) patients < 16 years old, and (4) insufficiency fractures in elderly patients. Included patients were two men and four women with an average age of 37.6 (range, 28–44) years, four cases of type B (two of type B2 and two of type B3) and two cases of type C (two of type C2) fractures using the Marvin Tile classification [3]. Among them, fracture mechanisms included traffic accidents (*n* = 1), falls from heights (*n* = 4), and crush injuries (*n* = 1). Preoperatively, all patients received a detailed neurological examination and a complete radiological evaluation, including anteroposterior (AP), pelvic inlet, and outlet views, and computed tomography (CT) scans of the pelvis to evaluate the displaced pelvic ring comprehensively. The surgery was scheduled as soon as the patients’ physiological condition was stable, with an average duration of 6.8 (range, 1–14) days from injury to surgery (Table 1).

**Table 1:** Patient characteristics

Patient	Age (years)	Sex	Tile type	Surgical procedures (anterior + posterior fixation)	Implant site of anterior ring	Time from injury to surgery (days)	Operation time (min)	Blood loss (mL)	Injury mechanism	Matta criteria	Complications
1	39	F	B2	MPSRF + PIS + SPF	U	14	151	200	Fall	Excellent	
2	44	F	B3	MPSRF + SPF	B	8	312	600	Fall	Good	
3	28	F	B2	MPSRF + PTP	U	8	194	100	Fall	Excellent	
4	37	F	C2	MPSRF + PTP + SPF	B	8	292	350	Fall	Excellent	
5	36	M	C2	MPSRF + PIS + SPF	U	2	183	160	Crush	Excellent	Rod breakage
6	42	M	B3	MPSRF	B	1	283	700	Traffic	Excellent	

Patient data as abbreviated terms, *F* female, *M* male, *MPSRF* modified pedicle screw-rod fixation, *PIS* percutaneous iliosacral screw, *PTP* percutaneous transiliac plate, *SPF* spinal pelvic fixation, *U* unilateral, *B* bilateral

### Surgical technique

**Fig. 1:**
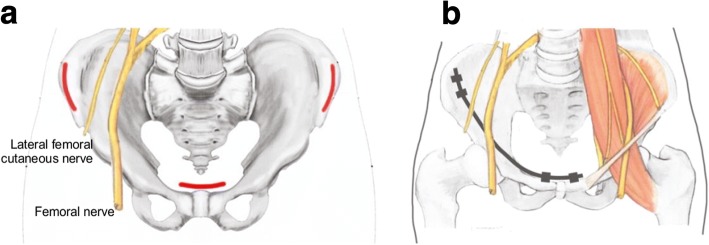
**a** Incisions for minimally invasive modified pedicle screw-rod fixation (MPSRF). This includes a curved incision over 1 cm proximal to the anterior inferior iliac spine (AIIS) and a transverse incision one fingerbreadth superior to the pubic symphysis. Lateral femoral cutaneous nerve coursing medially and inferiorly to the iliac incision. Red line: skin incisions, yellow line: lateral femoral cutaneous nerve (LFCN) and femoral nerve. **b** The final construct of MPSRF with the pedicle screws placed over the iliac wing and superior pubic ramus. Rod was placed submuscularly under the major neurovascular bundles. There are at least a few centimeters between the screw and LFCN, to prevent compression through this region

All surgical operations were performed by one surgeon with the patients under general anesthesia. Patients were positioned on a radio-transparent operation table in the supine position. The skin was prepared and draped from above the umbilicus to the lower extremities to facilitate the reduction technique. The lateral window of the ilioinguinal approach started at 1 cm proximal to the anterior superior iliac spine (ASIS) and the posterior window extended along the iliac crest with approximately 4- to 5-cm-long curves (Fig. 1a). The origins of the abdominal and iliacus muscles at the iliac crest were sharply elevated within a limited area. The iliacus muscle from the inner table of the iliac wing was elevated using a blunt dissection, which continued medially to the superior pubic ramus. Flexing the hip was also helpful for relaxing the iliopsoas muscle when small sub-muscular tunnels were being created. Once the two appropriate entry points, 3 to 4 cm apart, were identified, the cortex was opened using a 2.5-mm drill bit at approximately 4 cm medial to ASIS for establishing the bony corridors. Two 4.0-mm-diameter Axon Spine System (Depuy Synthes, Switzerland) polyaxial pedicle screws were inserted in the same direction (Fig. 1b). The length of the screws varied from 12 to 20 mm, depending on the habitus of the patient. The procedure was repeated for the contralateral hemipelvis if bilateral anterior pelvic fractures were present.

Another transverse Pfannenstiel incision began at one fingerbreadth proximal to the pubic symphysis and was extended laterally for approximately 4 to 5 cm (Fig. 1a). Sharp dissection was performed on the anterior rectus fascia, and the subcutaneous fatty layer was elevated away from the rectus fascia. The rectus abdominis muscle was split along the linea alba, and the transversalis fascia was opened just proximal to the pubic symphysis to allow access to the retropubic space of Retzius. The bladder was mobilized bluntly from the anterior pelvic ring. The insertion of the rectus abdominis muscle was left intact on the anterior aspect of the pubic rami but was released on the superior border of the pubic rami and symphysis. A sub-muscular plane was created using a blunt dissection along the cranial surface of the superior ramus to the iliac wing where the sub-muscular tunnel from both incisions connected. The placement of the pedicle screws on the ipsilateral or contralateral pubic ramus was determined by the fracture pattern. If pubic symphysis diastasis occurred or the residual fragment of the ipsilateral pubic ramus was not large enough for the placement of the two pedicle screws, screw anchoring to the contralateral pubic ramus was necessary. After the two starting points on the superior plane of the superior ramus were confirmed radiographically, the cortex was opened with a 2.5-mm drill bit to establish the bony corridor towards the inferior ramus. Next, two 3.5-mm-diameter polyaxial pedicle screws were inserted (Fig. 1b), and safe placement of screw positions was confirmed using the pelvic inlet view. The screw length varied from 20 to 40 mm.

**Fig. 2:**
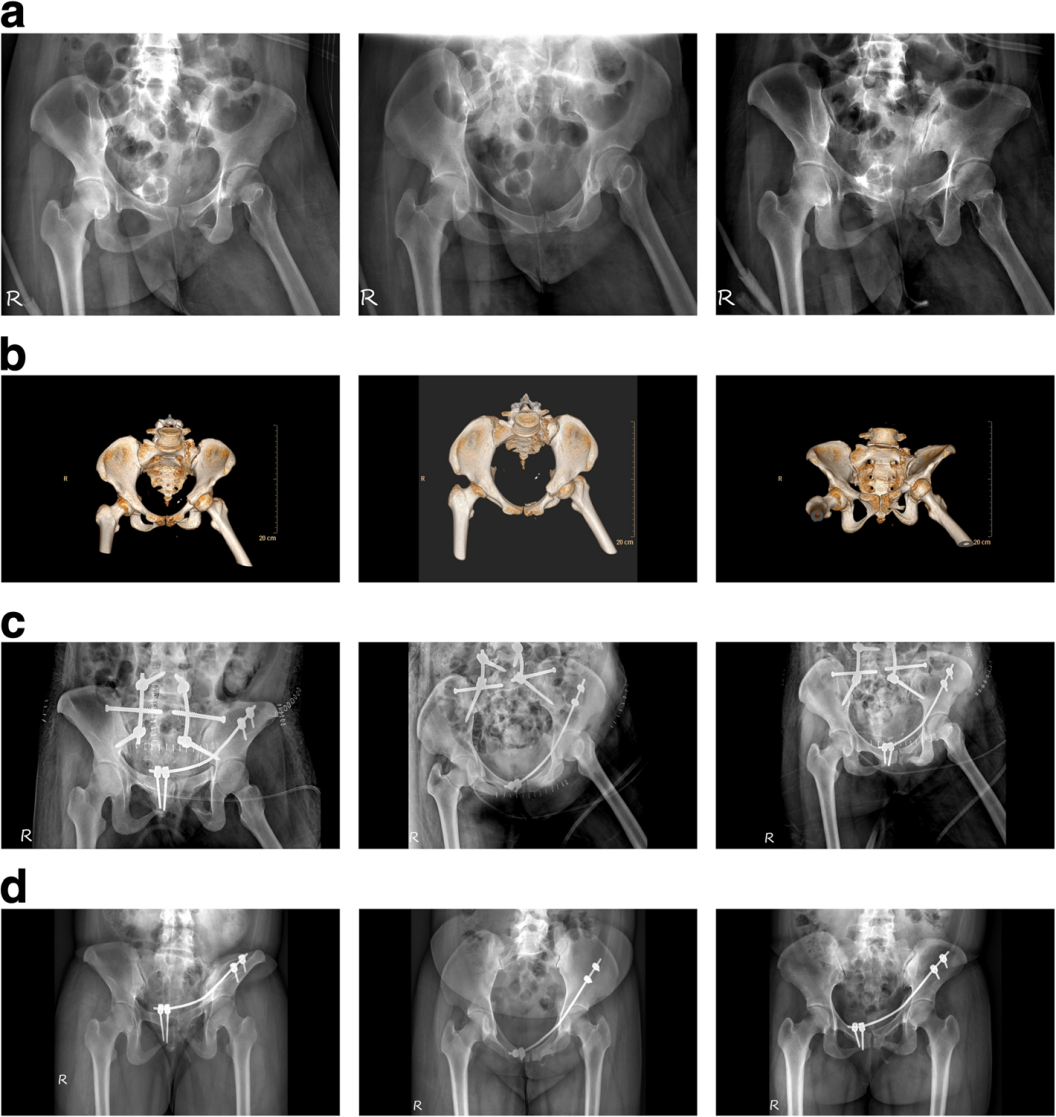
A 39-year-old woman with anterior and posterior pelvic ring injuries caused by a fall. **a** Preoperative pelvic radiology series (AP, inlet, outlet view) demonstrating left superior and inferior pubic ramus fractures combined with a sacral fracture. **b** Preoperative 3D reconstructed CT images (AP, inlet, outlet view). **c** Postoperative pelvic radiology series (AP, inlet, outlet view) demonstrating percutaneous iliosacral screws, spinal pelvic fixation, and the modified pedicle screw-rod fixation. **d** Postoperative pelvic radiology series (AP, inlet, outlet view) at 22 months follow-up, demonstrating bone union

**Fig. 3:**
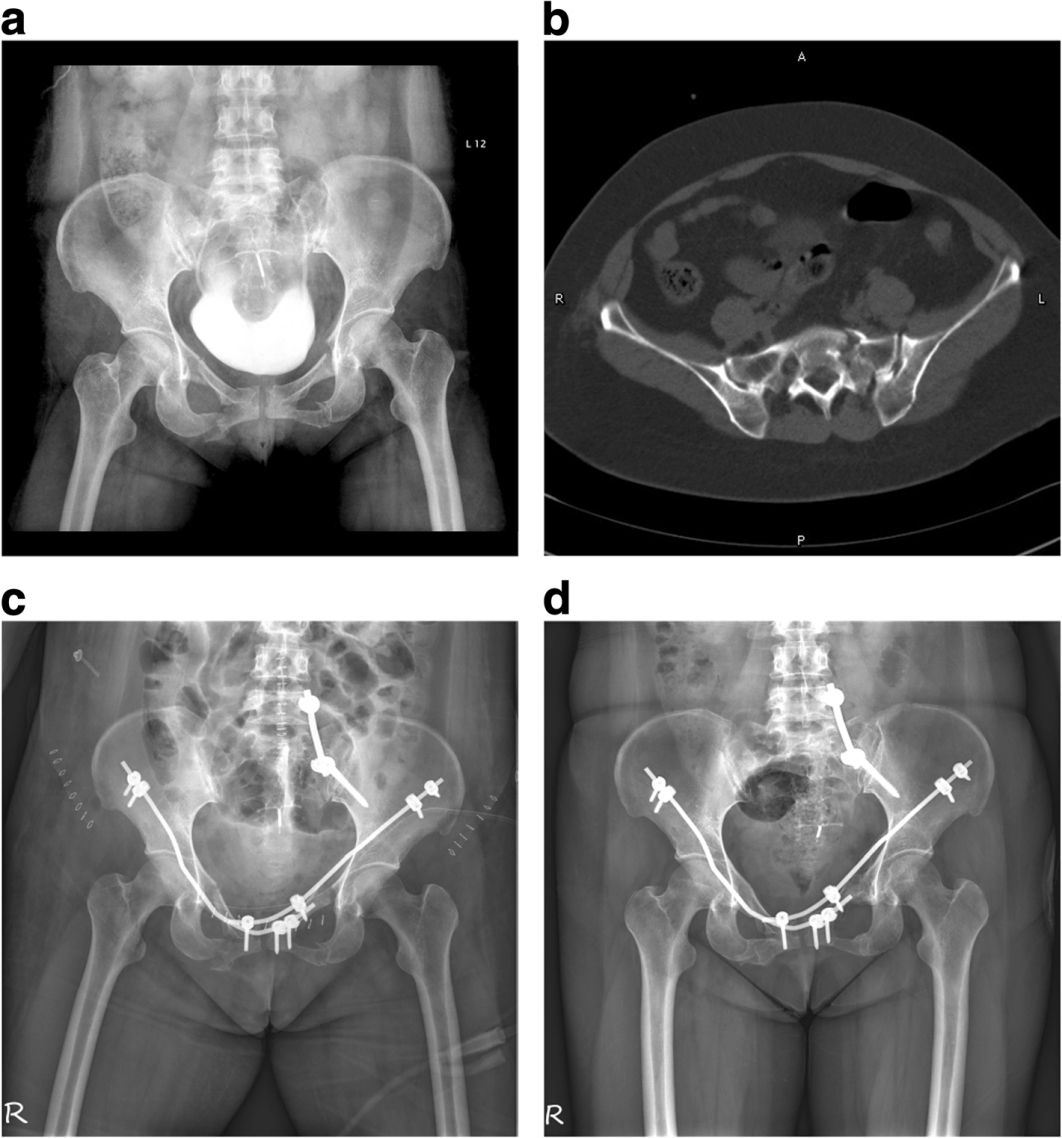
A 44-year-old woman with anterior and posterior pelvic ring injuries caused by a fall. **a** Preoperative radiology plain images showing bilateral pubic rami fracture. **b** Preoperative axial computed tomography scan image showing a left side sacral fracture. **c** Postoperative radiology plain images showing good reduction with the modified pedicle screw-rod fixation technique for the anterior ring and spinal pelvic fixation for the sacral fracture. **d** Radiology plain image showing fracture healing at 8 months postoperatively

Once both side screws were in place and an acceptable reduction had been achieved, a rod template was placed on the pedicle screws through the submuscular tunnel to estimate its length and curvature. Next, the contoured rod (3.5 mm diameter) was gently inserted in the submuscular tunnel from the incision at the pubic ramus to the iliac wing below the iliacus and psoas muscles. The rod was connected to pedicle screw heads, whose caps were loosely secured to maintain the rod in place. At this point, reduction tools were used to manipulate the fracture site into an appropriate reduction, and the screw caps were locked with a torque screwdriver to maintain the reduction. Radiographs of typical cases are provided to illustrate the preoperative and postoperative changes (Figs. 2 and 3). Suitable reduction and implant position were confirmed on the C-arm fluoroscopic AP, inlet, and outlet views. Prophylactic intravenous antibiotics were administered until 24 h postsurgery to prevent infections.

### Postoperative management and follow-up

Non-weight-bearing functional exercises of the lower limbs and joints were initiated on the postoperative day 1. Patients were allowed to sit on the bedside at 1 week, and crutch-assisted partial weight bearing was allowed from 6 weeks, postoperatively. They were allowed to walk with full weight-bearing at 8 weeks, postoperatively.

Routine follow-ups for clinical and radiological assessment were scheduled for postoperative weeks 4 and 8; months 3, 6, and 9; and at 1 year. Radiographic images at each follow-up visit included a three-view pelvis series (AP, inlet, and outlet). At all visits, thorough neurological examinations focused on LFCN and the femoral nerves, physical examinations focused on pelvic stability, and local irritation of the implants was performed.

### Outcome measures

Outcome measures were total operation time, blood loss, complications, reduction achieved from surgery, and fracture healing time. Radiographs were assessed by a specialist surgeon with experience in pelvic surgery, who was blinded to all identifiable patient information. The results of fracture reduction were graded based on published criteria [16] as excellent (0–5 mm), good (5–10 mm), fair (10–20 mm), and poor (> 20 mm), according to the maximal residual displacement of the fracture site in the three-view pelvis series. Fracture healing was determined by the progression of callus formation until radiographic union and by the ability of the patient to bear weight without pain. Failure of fixation was assessed by implant breakage, uncoupling of the instruments, or by loosening at the screw-bone interface.

Specific complications for our technique included injuries of LFCN, the femoral artery, femoral vein, femoral nerve, and the round ligament in women or the spermatic cord in men. Moreover, other general complications included infections, erosion of the soft tissue overlying the implant, loss of fracture reduction, implant failure, nonunion, and heterotopic ossification.

## Results

In our series, one patient underwent anterior fixation using MPSRF alone and five patients underwent both anterior and posterior fixations. These five patients included two patients with percutaneous iliosacral screws and spinal pelvic fixations, one patient with a percutaneous transiliac plate and spinal pelvic fixation, one patient with a percutaneous transiliac plate, and one patient with spinal pelvic fixation. Three patients underwent unilateral anterior ring fixation, and the other three patients underwent bilateral fixation. The average operative time and mean intraoperative blood loss for unilateral anterior ring fixation were 176.0 (range, 151–194) min and 153.3 (range, 100–200) mL, and 295.6 (range, 282–312) min and 550.0 (range, 350–700) mL for bilateral anterior ring fixation. The mean time of injury-to-surgery was 1 day in the patient who underwent anterior pelvic ring fixation alone and 8 (range, 2–14) days in patients who underwent both anterior and posterior fixation (Table 1).

No iatrogenic neuropraxia of LFCN or femoral nerve palsy occurred after the surgeries. No intraabdominal hollow organ injury or urinary bladder injury was observed in relation to screw insertion. In addition, no patient experienced postoperative complications such as hemorrhagic shock, deep venous thrombosis, or wound infections.

All patients were followed up for an average of 26.7 (range, 14–40) months, and no one died or was lost to follow-up. During the follow-up period, healing was achieved in all pelvic fractures at a mean postoperative period of 4 (range, 3–6) months. Fracture reduction was excellent in five patients and good in one patient, postoperatively (Table 1). No loss of reduction, delayed osseous union, nonunion, malunion, loss of fixation, loosening of implant, or heterotopic ossification was observed in physical and radiographic examinations during the follow-up period. One patient suffered from a broken connecting rod at 4 months postsurgery, but complete fracture healing without discomfort was noted.

All the patients could sit normally, stand, squat, and lie in the prone position or on either side and were reintegrated into society without any restrictions. The implant was removed in one patient, 8 months postoperatively, at the patient’s request. Five patients preferred to retain the device and have reported no problems to date.

## Discussion

Successful management of unstable pelvic fractures remains challenging for orthopedic surgeons, and the optimal fixation technique remains controversial. To combine the advantages of the pedicle screw-rod system and the pelvic bridge techniques for treating unstable anterior pelvic ring fractures, we designed a new form of minimally invasive pelvic fixation using pedicle screw-rod fixators, which were applied submuscularly from the iliac wing to the superior pubic ramus. This method can be used for patients with residual instability in the anterior ring after the posterior pelvis has either been fixed or verified to be stable by stressing the pelvis intraoperatively under fluoroscopy.

The concept of minimally invasive plate osteosynthesis in pelvic fractures has recently been introduced. Yu et al. introduced a minimally invasive plate osteosynthesis technique for pubic ramus fracture treatment [17]. Owing to the lack of direct visualization, some anatomic structures, including LFCN, the femoral artery, femoral vein, femoral nerve, and the round ligament in women or the spermatic cord in men, are theoretically at risk of injury during implant placement. LFCN irritation is the most prevalent iatrogenic neurovascular complication during surgical treatment of anterior ring fractures. Temporary LFCN neuropraxia was observed by Vaidya et al. [18] in up to 30% of the 91 patients included in their study. A previous study verified that placement of implants over AIIS could be complicated with LFCN injury and hip joint capsule violation [19]. Furthermore, INFIX pedicle screw placement requires deep dissection in the space between the sartorius and the tensor fasciae latae muscles where LFCN is vulnerable. Hence, we changed the pedicle screw positions of the ilium from the AIIS, a high-risk zone for LFCN, to the inner table of the iliac wing medial to the ASIS level, which is further away from LFCN (Fig. 1b). Theoretically, the selected locations of the pedicle screws were relatively safe areas for surgical dissection, implant application, and removal. However, with a 2.9 to 4% incidence of unusual superolateral course, the nerve may be endangered during dissection over the anterior aspect of the iliac wing when the attachment of the pelvic bridge construct is contemplated [8, 15]. To avoid potential impingement on LFCN, medial and lateral fixation of the implant should be performed under direct visualization and after careful dissection [20].

In previous bridge techniques, the subcutaneous corridor was created directly from the iliac crest to the pubic tubercle with a high risk of neurovascular injury and abdominal perforation. The connecting rods of the pedicle screws in the MPSRF technique were fixed through the submuscular tunnel, below the vital neurovascular bundles, as mentioned above. Alternatively, Hoskins et al. [21] attributed traction-induced neuropraxia to the large size of lumbar pedicle screws but the implants used in the MPSRF technique are small cervical pedicle screws. Unlike other studies, there were no cases of postoperative temporary LFCN neuropraxia in our series; this might be explained by the changes in screw positions, the application method of the connecting rod, the smaller diameter of screws, and our meticulous approach during dissection because of our awareness of this complication based on previous studies.

A cadaveric study found that the femoral nerve is at the greatest risk of compression by the rod [22]. In the MPSRF technique, the connecting rod was placed under the muscular and neurovascular structure; therefore, the compression force of the femoral nerve could be minimized, thus decreasing the risk of femoral nerve injury. In our series, no femoral nerve palsy was noted during the follow-up period. Theoretically, the MPSRF technique is relatively protective of nerves compared to other subcutaneous techniques for anterior pelvic ring injuries. One of the limitations of our study was the small sample size and the limited follow-up duration, which might explain the absence of neurovascular complications. Future studies with larger sample sizes and longer follow-up periods may reveal the actual incidences of the complications related to the MPSRF technique.

In the INFIX technique, large-diameter lumbar spinal pedicle screws, ranging from 6.5 to 7.5 mm, were used for fixation [23]. In the MPSRF technique, we used small-diameter pedicle screws, ranging from 3.5 to 4.0 mm, which are used in cervical spinal surgery. While Owen et al. [24] reported fixation failure with small-diameter screws salvaged by larger screws in morbidly obese patients, we increased the number of screws in the MPSRF technique for fixation of the anterior pelvic ring. Two to three pedicle screws were used in the INFIX technique, while four screws were used in MPSRF. The thinner screw head of smaller pedicle screws not only prevented soft tissue irritation, it also preserved more space for adjacent screw placement. Although Vigdorchik et al. pointed out that anterior neutralization plate fixation is stiffer than INFIX for fracture stability at the pubic symphysis [25], the lack of a biomechanical study that compared fixation stability between MPSRF and plate osteosynthesis limited our study. Based on our results, we believe that smaller-diameter and more pedicle screws could provide suitable and acceptable fixation stability in anterior pelvic ring fractures without loss of reduction, osseous nonunion, or loosening of screws during the follow-up period, although biomechanical testing is required for verification.

The type of pedicle screw determines the performance of fixation stability, with monoaxial screws providing significantly greater stiffness than polyaxial screws [26]. However, the polyaxial screws used in the MPSRF technique reduced the difficulty of rod manipulation. The use of a monoaxial screw by an inexperienced surgeon may be challenging because the accurate placement of screws is mandatory for precise positioning of the connecting rod. Conversely, polyaxial screws allow inaccuracies in their placement. Further biomechanical studies on the MPSRF fixation device are necessary to verify our results.

Previous studies have shown that subcutaneous devices are typically palpable in the lower abdominal fold, and surgeons recommend implant removal at a mean postoperative period of 1.5 to 9.4 months once the patient’s injuries have healed and their symptoms have plateaued [27]. However, the submuscular device in the MPSRF technique was not palpable and did not require removal in the absence of complications.

This study has some noteworthy limitations. First, the small sample size and lack of long-term follow-up warrant future multicenter prospective studies for final evaluation. Second, our analysis was based on clinical cases in the prediction of the stability of the fixation technique, and a biomechanical study is necessary for more convincing conclusions. Third, this study does not report functional outcomes, and a long-term functional score analysis is necessary. Fourth, the MPSRF technique used US Food and Drug Administration-approved implants for cervical spinal fixation for an unapproved method in the pelvis. It is thus an off-label use.

## Conclusions

The operative time of the MPSRF technique was relatively longer than that in previous subcutaneous techniques, which might be explained by the deeper approach of dissection, the more complex implants used, and bilateral anterior pelvic ring fractures. The blood loss in our series was not as low as that in other subcutaneous techniques, which might be due to the open direct reduction methods we employed and the longer surgical time of the MPSRF technique.

The MPSRF technique afforded satisfactory clinical and radiological outcomes with fewer complications in the present study. We believe that the modified technique represents a novel minimally invasive procedure for the treatment of anterior pelvic ring instability and offers a reliable and effective alternative to current surgical techniques.

## Abbreviations

AIIS: Anterior inferior iliac spine
ASIS: Anterior superior iliac spine
CT: Computed tomography
LFCN: Lateral femoral cutaneous nerve
MPSRF: Minimally invasive modified pedicle screw-rod fixation

## References

[1] Giannoudis PV, Pape HC (2004). Damage control orthopaedics in unstable pelvic ring injuries. Injury.

[2] Bi C, Wang Q, Nagelli C, Wu J, Wang Q, Wang J (2016). Treatment of unstable posterior pelvic ring fracture with pedicle screw-rod fixator versus locking compression plate: a comparative study. Med Sci Monit.

[3] Tile M (1988). Pelvic ring fractures: should they be fixed?. J Bone Joint Surg. (Br).

[4] Starr AJ, Nakatani T, Reinert CM, Cederberg K (2008). Superior pubic ramus fractures fixed with percutaneous screws: what predicts fixation failure?. J Orthop Trauma.

[5] Tucker MC, Nork SE, Simonian PT, Routt ML (2000). Simple anterior pelvic external fixation. J Trauma.

[6] Vaidya R, Colen R, Vigdorchik J, Tonnos F, Sethi A (2012). Treatment of unstable pelvic ring injuries with an internal anterior fixator and posterior fixation: initial clinical series. J Orthop Trauma.

[7] Mason WT, Khan SN, James CL, Chesser TJ, Ward AJ (2005). Complications of temporary and definitive external fixation of pelvic ring injuries. Injury.

[8] Cole PA, Gauger EM, Anavian J, Ly TV, Morgan RA, Heddings AA (2012). Anterior pelvic external fixator versus subcutaneous internal fixator in the treatment of anterior ring pelvic fractures. J Orthop Trauma.

[9] Hiesterman TG, Hill BW, Cole PA (2012). Surgical technique: a percutaneous method of subcutaneous fixation for the anterior pelvic ring: the pelvic bridge. Clin Orthop Relat Res.

[10] Gardner MJ, Mehta S, Mirza A, Ricci WM (2012). Anterior pelvic reduction and fixation using a subcutaneous internal fixator. J Orthop Trauma.

[11] Scheyerer MJ, Zimmermann SM, Osterhoff G, Tiziani S, Simmen HP, Wanner GA (2014). Anterior subcutaneous internal fixation for treatment of unstable pelvic fractures. BMC Res Notes.

[12] Wu X, Liu Z, Fu W, Zhao S, Feng J (2017). Modified pedicle screw-rod fixation as a minimally invasive treatment for anterior pelvic ring injury: an initial case series. J Orthop Surg Res.

[13] Cole PA, Dyskin EA, Gilbertson JA (2015). Minimally-invasive fixation for anterior pelvic ring disruptions. Injury.

[14] Fang C, Alabdulrahman H, Pape HC (2017). Complications after percutaneous internal fixator for anterior pelvic ring injuries. Int Orthop.

[15] Doklamyai P, Agthong S, Chentanez V, Huanmanop T, Amarase C, Surunchupakorn P (2008). Anatomy of the lateral femoral cutaneous nerve related to inguinal ligament, adjacent bony landmarks, and femoral artery. Clin Anat.

[16] Matta JM (1996). Indications for anterior fixation of pelvic fractures. Clin Orthop Relat Res.

[17] Yu X, Tang M, Zhou Z, Peng X, Wu T, Sun Y (2013). Minimally invasive treatment for pubic ramus fractures combined with a sacroiliac joint complex injury. Int Orthop.

[18] Vaidya R, Kubiak EN, Bergin PF, Dombroski DG, Critchlow RJ, Sethi A (2012). Complications of anterior subcutaneous internal fixation for unstable pelvis fractures: a multicenter study. Clin Orthop Relat Res.

[19] Haidukewych GJ, Kumar S, Prpa B (2003). Placement of half-pins for supra-acetabular external fixation: an anatomic study. Clin Orthop Relat Res.

[20] Moazzam C, Heddings A, Moodie P, Cole PA (2012). Anterior pelvic subcutaneous internal fixator application: an anatomic study. J Orthop Trauma.

[21] Hoskins W, Bucknill A, Wong J, Britton E, Judson R, Gumm K (2016). A prospective case series for a minimally invasive internal fixation device for anterior pelvic ring fractures. J Orthop Surg Res.

[22] Apivatthakakul T, Rujiwattanapong N (2016). Anterior subcutaneous pelvic internal fixator (INFIX), Is it safe? A cadaveric study. Injury.

[23] Kuttner M, Klaiber A, Lorenz T, Füchtmeier B, Neugebauer R (2009). The pelvic subcutaneous cross-over internal fixator. Unfallchirurg.

[24] Owen MT, Tinkler B, Stewart R (2013). Failure and salvage of “INFIX” instrumentation for pelvic ring disruption in a morbidly obese patient. J Orthop Trauma.

[25] Vigdorchik JM, Esquivel AO, Jin X, Yang KH, Onwudiwe NA, Vaidya R (2012). Biomechanical stability of a supra-acetabular pedicle screw internal fixation device (INFIX) vs external fixation and plates for vertically unstable pelvic fractures. J Orthop Surg Res.

[26] Eagan M, Kim H, Manson TT, Gary JL, Russell JP, Hsieh AH (2015). Internal anterior fixators for pelvic ring injuries: do monaxial pedicle screws provide more stiffness than polyaxial pedicle screws?. Injury.

[27] Shetty AP, Bosco A, Perumal R, Dheenadhayalan J, Rajasekaran S (2017). Midterm radiologic and functional outcomes of minimally-invasive fixation of unstable pelvic fractures using anterior internal fixator (INFIX) and percutaneous iliosacral screws. J Clin Orthop Trauma.

